# Nasal Chondrocytes Intensively Invade and Repair Pathologically Altered Cartilage Through Intrinsic Genomic Mechanisms: A Narrative Review

**DOI:** 10.2174/0115733971359145250329194434

**Published:** 2025-04-09

**Authors:** Victoria A. Shestakova, Ekaterina I. Smirnova, Longfeng Rao, Ilya V. Kolobaev, Dmitry A. Atiakshin, Michael A. Ignatyuk, Mikhail E. Krasheninnikov, Bagavdin G. Ahmedov, Sergey A. Ivanov, Ilya D. Klabukov, Peter V. Shegay, Andrey D. Kaprin, Denis S. Baranovskii

**Affiliations:** 1 National Medical Research Radiological Center of the Ministry of Health of the Russian Federation, Koroleva st. 4, 249036, Obninsk, Russia;; 2 Obninsk Institute for Nuclear Power Engineering of the National Research Nuclear University MEPhI, Studgorodok 1, 249036, Obninsk, Russia;; 3 ETH Zurich, Rämistrasse 101, 8092, Zurich, Switzerland;; 4 Patrice Lumumba Peoples Friendship University of Russia (RUDN University), Mikluho-Maklaya st., 6, 117198, Moscow, Russia;; 5 National Medical Research Center for Surgery named after A.V. Vishnevsky of the Ministry of Health of the Russian Federation, Bolshaya Serpukhovskaya, 2115093, Moscow, Russia;; 6 University of Basel, Petersplatz 1, 4001, Basel, Switzerland

**Keywords:** Articular chondrocytes, cartilage, cell therapy, nasal chondrocytes, osteoarthritis, regenerative medicine

## Abstract

Articular cartilage, a crucial component of joint structure, ensures smooth articulation and efficient load distribution within the joint. However, its integrity is compromised in various pathological conditions, such as osteoarthritis, leading to significant alterations in its structure and function. This process was significantly correlated with Extracellular Matrix (ECM) degradation, loss of collagen type II, and increased expression of matrix metalloproteinases (MMPs), particularly MMP-13. The ability of chondrocytes to invade into the ECM in pathologically altered tissue leads to cartilage repair and regeneration, and becomes the basis of chondrocyte cell therapy. Furthermore, the altered mechanical properties of the ECM in diseased cartilage, alongside the upregulation of chemotactic factors, contribute to the enhanced migratory behavior of chondrocytes. Interestingly, chondrocytes invading the ECM displayed signs of phenotypic changes, such as increased proliferation and expression of markers associated with chondrocytes' intrinsic genetic properties. The invasion of chondrocytes into the ECM is a response to cartilage damage, possibly driven by an attempt to repair the degraded ECM, and varies in chondrocytes from different sources, *i.e.*, articular cartilage or nasal septum. Nasal chondrocytes highlight the increase of ACAN, SOX9, N-cadherin, COL2A expression and decrease of IL1B, CXCL8, and MMPs gene family expression, which could relate to their unique phenotype properties. However, this response may paradoxically contribute to the progression of cartilage pathology by disrupting the tissue architecture and promoting further degeneration. Our review highlights the endogenous genetic properties of nasal chondrocytes to invade and repair damaged cartilage, offering promising avenues for cartilage repair and regeneration.

## 1 INTRODUCTION

Articular cartilage is a highly specialized tissue that covers the ends of bones in synovial joints, providing a smooth, lubricated surface for joint movement and distributing loads to the underlying bone [1]. Despite its critical role in joint function, articular cartilage has a limited ability to repair itself, making injury and degenerative diseases such as osteoarthritis (OA) particularly problematic.

OA is increasingly recognized as a whole joint disease, affecting not only cartilage but also other articular tissues [2, 3]. Recent evidence suggests that OA is characterized by subchondral bone remodeling [4], meniscal degeneration [5, 6], inflammation and fibrosis of both the infrapatellar fat pad and synovial membrane [7]. All these biomechanical and inflammatory processes lead to the displacement of chondrocytes toward a hypertrophic state and changes in their metabolic activity, causing abnormal matrix production, increased enzyme activity, and synthesis of inflammatory mediators, which contribute to the progression of OA [8, 9]. Chondrocytes adapting to the environmental stress of OA alter their metabolism with mitochondrial dysfunction, altered lipid and amino acid metabolism, and a switch from oxidative phosphorylation to glycolysis, regulated by AMP-activated protein kinase (AMPK) and mechanistic target of rapamycin (mTOR) pathways [9, 10]. In addition to biochemical changes, OA causes changes in the mechanical spectrum of chondrocytes, which include decreased Young's modulus of pathologically altered chondrocytes, decreased chondrocyte stiffness and viscosity [11], altered mechanotransduction pathways and responses to mechanical stimuli [7, 12], membrane depolarization [13], and loss of phenotypic stability followed by a transition to a hypertrophic state [8].

Cartilage repair, particularly articular cartilage, remains a significant challenge in orthopedics and regenerative medicine due to its limited self-healing capacity [14]. Traditional treatments like conservative drug therapy, surgical joint replacement, physiotherapy, and physical therapy have significant limitations and risks [15], prompting research into tissue engineering approaches.

Since chondrocytes play a major role in the formation of normal cartilage [16], they are essential components in the development of methods for cartilage repair and regeneration using cell-based technologies. However, these methods often require the expansion of cell culture with chondrogenic potential [17], which is possessed by several cell types from differentiated cell categories, in particular articular chondrocytes derived from mesoderm (MDCs) and nasal chondrocytes derived from neural crest (NCDCs) [18, 17].

Articular chondrocytes (ACs) have been used in clinical practice for thirty years [19], and autologous chondrocyte implantation (ACI) has been the gold standard in the field of cartilage repair based on differentiated cells [17]. However, a new concept has emerged that emphasizes the importance and necessity of treating the whole joint as a holistic organ system rather than just repairing cartilage damage, new methods and technologies for cartilage repair have been developed, and research has focused on comparing the potential of articular and nasal chondrocytes (NCs) for cartilage engineering and identifying the best strategy.

The aim of this review was to describe the underlying sources of unique properties of NCs primarily caused by their internal genetic characteristics.

## 2 NASAL CHONDROCYTES AND ARTICULAR CHONDROCYTES FOR CARTILAGE REPAIR

**Fig. (1):**
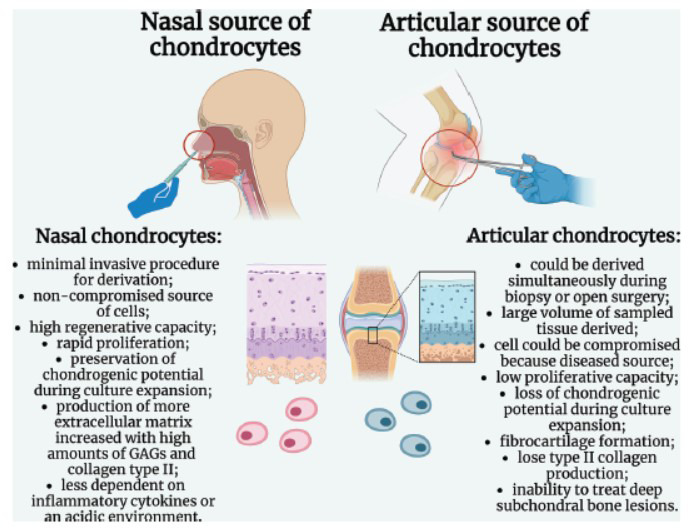
Nasal and articular sources of chondrocytes for cartilage repair. Created with Biorender.com.

As previously stated, ACs and NCs are two distinct types of cells that play crucial roles in the body's cartilage system, each with unique characteristics and functions. Both of these types of chondrocytes have found their application for regenerative medicine and orthopedics due to the limited self-healing capacity of cartilage (Fig. **1**).

ACs are the primary cells in articular cartilage, responsible for synthesizing and maintaining the extracellular matrix (ECM). Chondrocytes produce key ECM components, including collagen, proteoglycans, and non-collagen proteins, which contribute to cartilage's unique biomechanical properties [20]. ACs cells can be efficiently isolated from cartilage tissue obtained during surgical procedures, and their inherent ability to proliferate and maintain a chondrocytic phenotype *in vitro* has attracted considerable attention from researchers in the field of cartilage tissue engineering [21, 22]. When cultured in monolayers, AСs can rapidly proliferate, which makes them suitable for creating cell therapies aimed at repairing damaged cartilage. However, this strategy is fraught with difficulties and complications due to the unique properties of cartilage tissue and the complex biology of chondrocytes.

Firstly, as it was mentioned earlier, in the process of OA development, there are vague changes in the properties of chondrocytes. Derivation of such chondrocytes from pathologically altered cartilage mimics the cellular changes that may affect clinical outcomes [23]. It was observed that the mitochondrial respiratory rates were significantly reduced in chondrocytes derived from OA cartilage compared to those from non-osteoarthritic cartilage. These alterations were associated with lower cellular adenosine triphosphate (ATP) production, diminished mitochondrial membrane potential, and disrupted mitochondrial morphology [24]. Mitochondrial dysfunction plays a significant role in OA pathogenesis. It serves by amplifying the inflammatory response to cytokines, whereby reactive oxygen species (ROS) are produced and activate the nuclear factor kappa-light-chain-enhancer of activated B cells (NF-κB) pathway, contributing to cartilage degradation and synovial inflammation [25]. The use of such chondrocytes negates the possible positive effects of cell therapy.

Secondly, оne of the primary challenges in cartilage repair using chondrocyte-based cell therapy is the limited regenerative capacity of cartilage [26, 27]. Unlike other tissues, cartilage lacks the blood supply, lymphatic vessels, and nerves that are critical to the healing process. This avascular nature means that nutrients and repair cells cannot easily reach damaged areas, significantly slowing the repair process.

Thirdly, it was revealed that the isolation and expansion of ACs *in vitro* for re-implantation is another challenge. During the process of culturing, chondrocytes can begin to express genes more typical of fibroblasts, and this may result in the production of fibrocartilage, which is mechanically inferior to the hyaline cartilage found in articular surfaces [28]. Moreover, ACs, grown *in vitro* in a monolayer, become de-differentiated and lose type II collagen production and their potential for re-differentiation, which complicates their use in regenerative therapies [29]. They also have a lower proliferative capacity, which is partly why articular cartilage has a limited ability to repair itself after injury [30, 31].

Finally, ACs have been used in autologous chondrocyte implantation (ACI) and matrix-assisted chondrocyte implantation (MACI) techniques to repair damaged articular cartilage [32-34]. However, these approaches have limitations, including the need for two surgical procedures [35], limited availability of donor sites, long cartilage repair time [36-38], inability to treat deep subchondral bone lesions (more than 8 mm) [39] and focus on medium-sized defects rather than OA [40, 41].

It is also worth noting that the invasion of chondrocytes into the ECM is a response to cartilage damage, potentially driven by an attempt to repair the degraded matrix [42]. However, this response may paradoxically contribute to the progression of cartilage pathology by disrupting the tissue architecture and promoting further degeneration [28, 43].

Due to these limitations and adverse events, a new “nose-to-knee” (N2K) strategy has been developed that incorporates the use of NC for articular cartilage repair. NCs are the major cell type in the hyaline cartilage of the nasal septum and, like ACs, are capable of producing extracellular matrix proteins such as glycosaminoglycans and collagen. NCs have emerged as a promising cell source for cartilage repair and regeneration. NCs have shown a higher regenerative capacity when compared to ACs [18, 44-46]. Current research suggests that NCs can proliferate more rapidly and maintain their chondrogenic (cartilage-forming) potential even when expanded in culture, than ACs [47, 48]. Moreover, unlike ACs, NCs can be repeatedly de-differentiated and re-differentiated while retaining their cartilage-forming ability [47]. It was also noted that NCs produced more type II collagen and glycosaminoglycans, major components of the extracellular matrix of cartilage [49]. And NC-based engineered tissues show better integration with native cartilage and subchondral bone, and do not induce subchondral bone area increase associated with early OA, unlike AC-based grafts [50].

NCs have been explored in various clinical studies for their potential to repair cartilage defects beyond the nasal area, including the knee cartilage [51, 52]. The N2K strategy using NСs for articular cartilage repair has shown that harvesting NСs is much easier and has less risk of patient trauma than harvesting ACs, and the cells have much better biological potential and are much more resistant to the postoperative inflammatory environment [53].

Thus, application of NCs and tissue-engineered grafts grown on their basis could be a viable treatment for OA cartilage defects, as they maintain the cartilage matrix under inflammatory conditions, reduce the production of inflammatory and catabolic molecules by OA joint cells, and preserve cartilaginous features while integrating with native tissues in an *in vivo* OA environment [54].

## 3 CHARACTERISTICS OF NASAL AND ARTICULAR CHONDROCYTES IN OSTEOARTHRITIS HEALING

Cartilage degeneration in OA progresses gradually, characterized by a steady loss of collagen type II and a decline in mRNA expression of aggrecan (*ACAN*), SRY-box transcription factor 9 (*SOX9*), and collagen type II alpha 1 chain (*COL2A1*). The expression of WNT antagonists dickkopf WNT signaling pathway inhibitor 1 (*DKK1*) and frizzled related protein (*FRZB*) was lost, whereas hypertrophic markers such as *RUNX* family transcription factor 2 (*RUNX2*), collagen type X alpha 1 chain *COL10A1*, and Indian hedgehog signaling molecule (*IHH*) increase as OA progresses. Additionally, *DKK1* and *FRZB* levels negatively correlate with OA severity, while *RUNX2* and *IHH* exhibit a significant positive correlation with the disease's progression [55].

A comprehensive understanding of the genetic composition of NCs and ACs is essential for elucidating their functional characteristics and differentiation processes. NCs and ACs exhibit strong chondrogenic potential and express cartilage-specific markers, particularly collagen type II alpha 1 chain (*COL2A1*), collagen type IX alpha 1 chain (*COL9A1*), collagen type XI alpha 1 chain (*COL11A1*), aggrecan (*ACAN*), SRY-box transcription factor 9 (*SOX9*) and bone morphogenetic proteins (BMPs) [56].

Type II collagen is the primary component of hyaline cartilage, including nasal cartilage, providing structural support and tensile strength. It endows the cartilage with tensile strength and structural support, thereby ensuring its shape and integrity. The nasal septum contains more type II collagen and less type I and III collagens compared to other nasal cartilage regions, making it more hyaline in nature [57].

Type IX collagen is a fibril-bound collagen, typically present in cartilage alongside type II collagen. It endows the cartilage matrix with tensile strength and elasticity, which is of particular importance for the resilience of the cartilage structures in the nose. It acts as a mediator between chondrocytes and collagenous fibrils, thereby ensuring the maintenance of cartilage integrity and organization [58].

Type XI collagen plays a pivotal role in the maintenance of chondrocyte viability and functionality. Indeed, its expression has been demonstrated to impact chondrocyte proliferation, metabolic activity, and the synthesis of other extracellular matrix molecules, underscoring its pivotal role in maintaining cartilage homeostasis. Moreover, it plays a role in regulating the expression of other matrix proteins, such as type II collagen and aggrecan, thereby promoting the production of the cartilage matrix. Therefore, this ensures the optimal efficiency and health of the chondrocytes [59, 60].

Aggrecan is of critical importance to the ability of cartilage to resist compressive loads. It ensures the ability of cartilage to retain water, which is necessary for the maintenance of its elasticity and shock-absorbing properties. Aggrecan exerts a profound influence on the proliferation, differentiation, and metabolic activity of chondrocytes. The presence and quantity of aggrecan can influence the synthesis of other matrix components, thereby assisting in the maintenance of cartilage homeostasis [61, 62].

*SOX9* is a crucial transcription factor in chondrocyte development and function, regulating cartilage-specific genes and maintaining the chondrocyte phenotype. *SOX9* transcriptionally regulates the expression of cartilage-specific markers such as type II collagen and aggrecan, which, in turn, stimulate the synthesis of ECM components. *SOX9* is involved in complex molecular networks controlling its activity during chondrogenesis, including genetic regulation and post- translational modifications. Besides, *SOX9* participates in the repression of de-differentiation into a fibroblastic phenotype, which may be a consequence of pathological conditions or mechanical stress [63, 64].

BMPs exert a significant influence on the expression of crucial cartilage-specific markers, including *SOX9*, *COL2A1,* and Aggrecan. These factors are indispensable for the distinctive matrix composition of cartilage. BMPs stimulate the synthesis of extracellular matrix components, thereby contributing to the maintenance of the structural integrity and function of nasal cartilage. BMPs regulate the anabolic and catabolic activities of chondrocytes. They facilitate the synthesis of cartilage matrix proteins while inhibiting factors that promote cartilage degradation [65, 66].

The WNT signaling pathway plays a crucial role in chondrogenesis and OA pathogenesis. Endogenous expression of WNT antagonists dickkopf WNT signaling pathway inhibitor 1 (*DKK1)* and frizzled related protein (*FRZB)* is essential for chondrogenic differentiation and preventing hypertrophy. Inflammatory cytokines like interleukin-1 beta (IL1β) can disrupt this balance by downregulating *DKK1* and *FRZB* expression through nitric oxide signaling [67-69].

The Нomeobox (*HOX*) gene expression showed no significant differences between the NCs and ACs; however showed higher heterogeneity between donors. The involvement of *HOX* genes in the high regenerative and adaptive potential of NCs observed in previous studies could not be confirmed [70]. Articular chondrocytes appear to express more type II collagen and aggrecan than NCs [71].

### 3.1 Motile Activity of Nasal and Articular Chondrocytes

The capability of chondrocytes for invasion into the damaged cartilage matrix is underpinned by a complex interplay of genetic features, including specific genes and their promoters that regulate cellular behaviors essential for invasion [72]. Key genes implicated in this process include matrix metalloproteinases (MMPs), particularly MMP-1, MMP-2, and MMP-13, which are involved in the degradation of extracellular matrix components, facilitating chondrocyte migration. It has been shown that articular chondrocytes, for instance, express elevated levels of MMPs, especially MMP-3 and MMP-13, which enhance ECM degradation in conditions such as osteoarthritis [73]. In contrast, nasal chondrocytes exhibit lower expression of matrix-degrading enzymes like MMP-13 and a disintegrin and metalloproteinase with thrombospondin motifs 5 (ADAMTS5), which may limit their ability to penetrate damaged tissue [53].

The expression of these MMPs is closely regulated by various promoters and transcription factors, such as activator protein 1 (AP-1) and NF-κB, which respond to inflammatory cytokines and growth factors, influencing the overall migratory behavior of chondrocytes [74, 75]. Additionally, genes related to the Wnt/β-catenin signaling pathway, such as Wnt5a, have been shown to play a role in chondrocyte invasion, promoting cell motility and invasion through non- canonical signaling pathways [76, 77].

*SOX9*, a transcription factor critical for chondrogenesis, also influences the invasive capacity of NCs by regulating the expression of cartilage-specific matrix proteins and potentially modulating the expression of MMPs. Together, these genetic elements orchestrate a finely tuned regulatory network that enables NCs to invade and integrate into pathologically altered cartilage, highlighting their potential for therapeutic applications in cartilage repair [64].

The presence of interleukin-1 beta (IL-1β) significantly enhances the colonization intensity of chondrocytes and stimulates their migratory activity [78-80]. IL-1β achieves this by upregulating the expression of MMPs and reducing the synthesis of their natural inhibitors, the tissue inhibitors of metalloproteinases (TIMPs), in chondrocytes [81]. Furthermore, IL-1β stimulates the production of chemokines and adhesion molecules, enhancing the migratory capacity of chondrocytes towards the site of injury [82, 83].

The motile activity of chondrocytes, whether they are nasal or articular, is a complex process that involves several cellular mechanisms, including cell migration, proliferation, and ECM remodeling. Chondrocytes are the only cell type found in cartilage and are responsible for the maintenance of the cartilage extracellular matrix. While traditionally considered to be relatively immobile, recent studies have shown that chondrocytes can exhibit motile behavior, particularly during cartilage repair and in response to injury [84].

The migratory potential of ACs is constrained, and their capacity for repairing extensive cartilage lesions is limited. Furthermore, this capacity declines with age [85]. The rigid extracellular matrix that surrounds chondrocytes contributes to the restricted mobility of these cells [86].

In contrast to the migratory capacity of AСs, that of NСs is often observed to be increased. Research has identified a migratory subpopulation of NCs with progenitor cell characteristics, displaying a high migratory capacity and responsiveness to chemotactic stimulation, and can differentiate into osteogenic and chondrogenic lineages [87].

The migration potential of the chondrocytes is regulated by various genes and their encoded proteins. Ras homolog family member A (RhoA) is a small guanosine triphosphate (GTPase) that plays a crucial role in the regulation of the cytoskeleton, which is essential for cell motility. RhoA influences the formation of actin stress fibers and focal adhesion points, which are necessary for cell movement [88]. Similar to RhoA, Ras-related C3 botulinum toxin substrate 1 (Rac1) is another member of the Rho family of GTPases. It is involved in the control of the actin cytoskeleton and is crucial for lamellipodia formation at the leading edge of migrating cells, facilitating cell movement [89].

Another GTP-regulated enzyme, cell division cycle 42 (CDC42), is a member of the Rho GTPase family that regulates cell polarity and directional migration. It plays a role in the formation of filopodia, small, finger-like protrusions that help cells to sense their environment and direct their movement [90].

On the other hand, the expression of some membrane proteins could reduce the migration activity of chondrocytes, and play a negative role in selecting for cell invasion capabilities. Integrins are transmembrane receptors that facilitate cell-ECM adhesion. They play a critical role in transmitting signals between the ECM and the cytoskeleton, affecting cell migration and positioning [62]. Another membrane protein, CD44, is a cell surface glycoprotein involved in cell-cell and cell-matrix interactions. It has been shown to play a role in the migration of chondrocytes, particularly through its interaction with hyaluronic acid, a major component of the cartilage ECM [91, 92].

### 3.2 Proliferation Activity of Nasal and Articular Chondrocytes

NCs and ACs are specialized cells involved in the formation and maintenance of cartilage in the nose and joints, respectively [47]. These cells have distinct roles but share some common pathways in terms of proliferation and maintenance of cartilage tissue. Research indicates that NCs demonstrate higher proliferation rates and extracellular matrix production, particularly under low oxygen conditions, and exhibit greater chondrogenic potential and matrix-forming capacity than other cell types [93, 94]. ACs exhibit lower proliferative capacity compared to nasal chondrocytes [95]. This limitation is partly due to their specialized function in maintaining cartilage matrix in joints, where they exist in a hypoxic environment and exhibit low metabolic activity. ACs are more susceptible to age-related changes and OA, leading to decreased ability to proliferate and repair damaged cartilage. Their capacity for cartilage repair declines with age, associated with reduced responsiveness to growth factors and increased production of proinflammatory factors due to cell senescence [85]. The regulation of their proliferation involves a complex interplay of various genes, growth factors, and signaling pathways.

The *SOX9* gene is a transcription factor crucial for chondrocyte differentiation and proliferation. *SOX9* plays a significant role in regulating the expression of other cartilage-specific genes, including *COL2A1* and aggrecan, essential components of the cartilage matrix [96]. The *COL2A1* gene encodes the type II collagen protein, which is a major component of the cartilage extracellular matrix. Mutations in *COL2A1* can affect chondrocyte proliferation and lead to skeletal disorders [97]. *ACAN* is a critical component of the cartilage extracellular matrix, providing the tissue with its unique compressive properties. The *ACAN* gene encodes aggrecan, and its expression is regulated by *SOX9*. Aggrecan is essential for the proper function and proliferation of chondrocytes [98].

Growth factor receptors play a significant role in chondrocyte proliferation. Members of the fibroblast growth factors (FGF) family, particularly FGF2 (basic FGF), play a significant role in stimulating the proliferation of chondrocytes. FGF signaling pathways are involved in the regulation of cell division and growth in both NCs and ACs [99]. Insulin- like growth factor 1 (IGF1) is another growth factor that promotes chondrocyte proliferation. It acts through the IGF1 receptor to activate intracellular signaling pathways that lead to cell growth [100]. FGF2 promotes chondrocyte proliferation and has been shown to enhance the migratory capacity of chondrocytes. It acts by binding to its receptors on the cell surface, initiating a cascade of intracellular signaling that promotes cell movement [99]. Transforming growth factor-β (TGF-β) plays a crucial role in chondrocyte function and cartilage homeostasis. TGF-β promotes chondrocyte proliferation and differentiation through various mechanisms. It enhances gap junction formation *via* Smad3/Smad4 signaling, improving intercellular communication [101]. Furthermore, TGF-β overexpression promotes the expression of the chondrogenic gene SOX9 and inhibits hypertrophic and mineralization markers COL10A1 and MMP-13 [102].

Understanding the specific roles of these genes in chondrocyte proliferation can provide insights into the mechanisms of cartilage growth and regeneration, as well as the pathogenesis of cartilage-related diseases. It is important to note that the balance between proliferation and differentiation of chondrocytes is critical for healthy cartilage function, and dysregulation of these processes can lead to diseases such as OA.

## 4 CLINICAL APPLICATIONS OF NASAL CHONDROCYTES

Articular Cartilage Repair is one of the most promising applications of NCs for the repair of damaged articular cartilage, particularly in the knee. NCs are less-invasive, harvested, expanded in culture, and then implanted into the damaged area, where they can contribute to the regeneration of the cartilage [103]. This approach has been explored in clinical trials, showing potential for improving joint function and reducing pain (Table **1**).

As it can be seen from the table, the utilisation of NC-derived cells for cartilage regeneration is becoming a subject of growing interest, because they exhibit properties that are similar to those of chondrocytes and, in addition, the morbidity associated with the harvesting process is less than that which occurs with the harvesting of articular chondrocytes. The use of NCs for cartilage repair exhibits considerable potential across a range of medical applications. NCs are particularly well-suited to complex surgical reconstructions of nasal and tracheal defects, as well as the treatment of patellofemoral osteoarthritis and degenerative lesions of the mandibular condyles, showing good integration with surrounding tissues. NCs exhibit better proliferation and chondrogenic capacity than debrided knee chondrocytes, making them a more suitable cell source for autologous cartilage grafts.

## 5 FUTURE RESEARCH

Preclinical studies, as well as clinical trials, showed significant changes in the therapeutic potency of NCs and ACs, but genetic evaluation did not lead to finding statistically significant differences, only some minor genes. However, recently published studies show that the primary characteristics of NCs' advantage are associated with immunological responses. Overall, the assessment of immunological responses in cell transplantation and evaluating their efficiency remains an unexplored field of reconstructive surgery and regenerative medicine [111]. Essential cellular responses and capturing macrophages and mast cells in the area could compromise the outcomes [112] and potentially lead to mistaken decisions. Another way to alter the cells efficiently is by the senescent modification of chondrocytes during cultivation [49, 113]. The balance between pro-apoptotic cytokines like tumor necrosis factor α (TNF-α), which induce cell death, and anti-apoptotic cytokines like interleukin 4 (IL-4) and interleukin 10 (IL-10), which protect against apoptosis, is crucial in determining cell fate and has significant implications for cartilage damage and OA progression [114]. NCs demonstrate less potential to form senescent populations, and therefore induce the senescent niche and lead to senescent drift in affected tissues after transplantation [115].

**Fig. (2):**
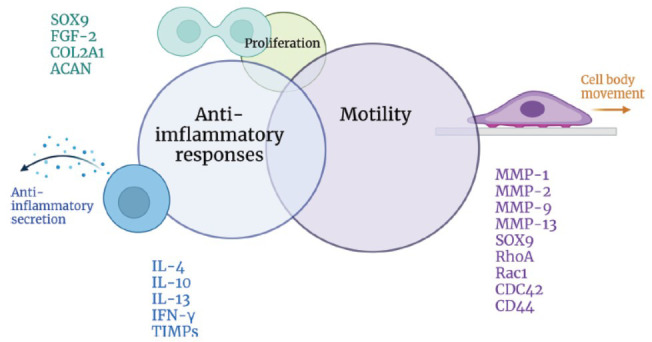
Preliminary model of the associations between pathways of chondrocyte motility, proliferation, and their anti-inflammatory responses. Created with Biorender.com.

Although there is currently no direct evidence for an association between chondrocyte motility and their anti-inflammatory secretion, we hypothesized that inner properties of chondrocytes are related to their proliferation as well as motility, and result in anti-inflammatory responses (Fig. **2**).

However, currently there is no sufficient evidence to prove this hypothesis; some separate studies showed a deeper association between cellular motility and proliferation, and inflamed tissue reparation. The case of NCs is a brilliant illustration of this thought. It has been proven that chondrocytes in monolayer culture exhibit migratory behavior and chemotaxis in response to fibroblast growth factor and hyaluronic acid. In joints affected by arthritis, the synthesis of high levels of fibroblast growth factor β (FGF-β) by inflamed cells creates an environment conducive to the initiation of directed chondrocyte migration and reparative processes [116]. In 3D models, FGF-β has been proven to promote the proliferation, migration, and differentiation of chondrocytes, particularly when they are present in a type II/I collagen matrix [117]. In the presence of inflammatory processes that simulate an injured joint, NC-based constructs exhibit enhanced recuperation from interleukin-1β deprivation in comparison to AC-based tissues [79]. These findings contribute to our understanding of chondrocyte behavior and may guide the development of improved strategies for cartilage tissue engineering and repair. We can also hypothesize that stimulation of motility-related genes in chondrocytes will contribute to their therapeutic efficacy. Cell motility is a fundamental principle in the processes of development and regeneration [118]. Rac1 is responsible for the regulation of cell polarisation and orientation during directed cell migration and proliferation [119]. It has been confirmed that insufficient expression of motility-related genes ENAH, actin regulator (*ENAH*), vasodilator-stimulated phosphoprotein (*VASP*), paxillin (*PXN*) and MMP14 contributes to reduced Rac1 activation in microtia chondrocytes [120].

At present, there are several strategies for delivering chondroregenerative genes to chondrocytes, which are being explored using viral and non-viral vectors [121]. Key targets include *SOX9*, α2-macroglobulin (A2M), which prevents ECM degradation, and a number of growth factors and transcription factors that enhance chondrogenesis and promote maintenance of the chondrogenic phenotype [122]. A2M is a protease inhibitor that has been demonstrated to promote cell migration, bind cytokines and growth factors, and enhance immune cell function. In the context of OA, A2M has demonstrated efficacy in slowing cartilage degeneration and reducing intra-articular inflammation in animal models [123, 124]. Current studies into optimising gene transfer methods are focused on striking a balance between efficacy and safety [125]. However, further research is needed to fully understand their biological properties and optimize manufacturing conditions to ensure safety and therapeutic potency in clinical applications.

## 6 DISCUSSION

NCs have emerged as a promising cell source for cartilage and bone regeneration due to their neural crest origin and unique properties. Studies have shown that NCs maintain proliferative capacity and produce extracellular matrix components under various culture conditions [93]. They can be used to engineer hypertrophic cartilage grafts that remodel into bone *in vivo*, demonstrating potential for maxillofacial reconstruction [126]. NCs exhibit adaptability to heterotopic transplantation sites and have been clinically used for nasal alar lobule reconstruction and articular cartilage repair [127]. Compared to other cell sources like AC, NCs offer advantages such as easier harvesting, reduced donor site morbidity, and a stable phenotype [46].

NCs can be directly used to repair cartilage that matches the complex shapes needed for nasal reconstruction, providing a more natural outcome than synthetic materials [128, 129]. This application is particularly relevant given the source of the cells, making them an ideal choice for nasal and septal surgeries. The obtained results demonstrate the promising use of NCs for the reconstruction of the alar lobule. It was shown that tissue-engineered constructs seeded with NCs allow to obtaining of stable and functional cartilage tissue with high chondrogenic activity.

The evaluation of the efficacy of treating patellofemoral OA with autologous nasal chondrocyte tissue-engineered cartilage (N-TEC) showed that the treatment of patients with OA with nasal chondrocyte-based tissue-engineered cartilage and NC-cell activated matrix (N-CAM) showed that the elastic modulus of N-TEC was 12 times higher compared with N-CAM. The ability of N-TECs to withstand the inflammatory conditions associated with knee OA has been demonstrated [130]. A transcriptome analysis identified that NCs have the capacity to inhibit the Wnt signaling pathway, which is responsible for the production of MMPs and the subsequent destruction of cartilage. This explains their resistance to inflammatory conditions [71]. Therefore, it was demonstrated that the implanted cells of NCs were capable of integrating, maintaining the structure and regenerating cartilage tissue in areas affected by OA.

It's also been shown that autologous nasal chondrocytes can be employed in the treatment of degenerative lesions of the mandibular condyle in the context of orthognathic surgery [131]. The biological characteristics of the nasal septal and mandibular condyle cartilages are distinct. The proliferation and synthesis of cartilage matrix in NCs are performed in differentiated chondrogenic cells, in contrast to chondrocytes in the mandibular condyle, whose secondary proliferation is performed in undifferentiated mesenchymal cells [132, 133]. The NCs were found to be characterized by a greater expression of aggrecan, Sox-9, N-cadherin and type II collagen, which has a significant impact on the mechanical properties of cartilage. This provides enhanced protection against pressure and the influence of other local mechanical factors, tensile strength and stretching ability, and supports the middle structure of the surface, in comparison to chondrocytes in the mandibular condyle [134, 135].

Tracheal reconstruction with NCs has been explored for their chondrogenic potential. Patients with tracheal defects or stenosis (narrowing of the trachea) can potentially benefit from tissue-engineered tracheal replacements [136, 137]. NCs, seeded onto biodegradable scaffolds, can be used to create new tracheal tissue that can be implanted into patients, offering a promising alternative to traditional grafts or synthetic replacements [138]. Application of NCs demonstrates a superior and more consistent proliferative and tissue-forming capacity in comparison to autologous chondrocytes [139].

However, there are limitations to the use of NCs, which may have an impact on their effectiveness in clinical applications and research. The potential for utilising cartilage grafts derived from the nasal septum is constrained by the quantity of nasal chondrocytes, in addition to donor site morbidity and surgical complications. Furthermore, a comprehensive investigation into nasal chondrocyte culturing methodologies is still necessary. For instance, there is a debate surrounding the impact of hypoxia conditions, which are characteristic of the articular cartilage environment, on nasal chondrocyte culturing [140, 141]. The successful resolution of these problems may facilitate a more effective utilisation of cartilage grafts. It is crucial to contemplate and implement minimally invasive techniques to diminish the risks associated with donor sites and enhance the safety of the procedures. Despite the advances made in scaffold design and nanomaterial science, which have enhanced tissue engineering techniques, significant challenges remain in attaining mechanical properties that are comparable to those of native cartilage and ensuring long-term biointegration.

Even so, evidence suggests that the use of chondrocytes derived from the nasal septum may represent a promising approach to the treatment of degenerative temporomandibular joint disorders.

## CONCLUSION

NCs, which are cells derived from the cartilage in the nose, have shown promising applications in the field of regenerative medicine and tissue engineering. Their ability to proliferate and differentiate into cartilage makes them an attractive source for repairing or regenerating damaged cartilage in various tissues. NCs exhibit distinctive regulatory mechanisms that allow for enhanced proliferative and migratory properties, which enhance their invasion into a cartilage scaffold. Because cells are derived from undamaged tissue, the resulting chondrocytes retain their functional properties, potentially leading to superior regenerative outcomes. The cell-derivation process is characterized by minimally invasive surgical procedures for biomaterial harvesting, thereby minimizing patient morbidity and increasing the feasibility of the procedure.

## AUTHORS’ CONTRIBUTIONS

Conceptualization, I.D.K., VAS; investigation, VAS, I.D.K., E.I.S.; data curation, I.D.K.; writing—original draft preparation, VAS, E.I.S., I.D.K.; writing—review and editing, VAS, E.I.S., I.D.K., D.S.B., L.R., I.V.K., D.A.A., M.A.I., M.E.K., B.G.A.; supervision, I.D.K., D.S.B., S.A.I., P.V.S., A.D.K. All authors revised and approved the manuscript.

## Figures and Tables

**Table 1:** Clinical applications of nasal chondrocytes.

**NCT Number**	**Study Title**	**Study Status**	**Conditions**	**Interventions**	**Phases**	**Enrollment**	**Study URL**
NCT04633928	Nasal Septum Perforation Treatment Using Tissue-Engineered Cartilage Graft	Completed	Nasal Cartilage Septum Perforations	BIOLOGICAL: N-TEC: Autologous nasal chondrocytes and ECM proteins	Phase 1	5	[104]
NCT02673905	Clinical Trial for the Regeneration of Cartilage Lesions in the Knee	Completed	Tear; Knee, Cartilage, Articular	OTHER: Tissue Engineered Product	NА	108	[105]
NCT03137914	Nasal Septum Autologous Chondrocytes Transplantation for Condylar Resorption After Orthognathic Surgery	Completed	Temporomandibular Joint Disorders	BIOLOGICAL: Autologous chondrocyte transplantation|PROCEDURE: orthognathic surgery	Phase 1	10	[106]
NCT06163573	Treatment of Patellofemoral Osteoarthritis With Engineered Cartilage.	Recruiting	Patellofemoral Osteoarthritis	BIOLOGICAL: N-TEC|BIOLOGICAL: Platelet rich plasma	Phase 2	75	[107]
NCT06576583	Implantation of Engineered Cartilage Grafts for Treatment of Patellofemoral Osteoarthritis *versus* Surgical Comparators.	Not yet recruiting	Patellofemoral Osteoarthritis	BIOLOGICAL: Engineered cartilage graft (N-TEC)|PROCEDURE: Autologous Matrix Induced Chondrogenesis (AMIC)|PROCEDURE: Patellofemoral Arthroplasty (PFA)	Phase 2	150	[108]
NCT01242618	Tissue Engineered Nasal Cartilage for Reconstruction of the Alar Lobule	Completed	Skin Carcinoma	BIOLOGICAL: engineered nasal cartilage graft	Phase 1	5	[109]
NCT01605201	Tissue Engineered Nasal Cartilage for Regeneration of Articular Cartilage	Completed	Cartilage Lesion|Degenerative Lesion of Articular Cartilage of Knee	BIOLOGICAL: Tissue-engineered cartilage graft	Phase 1	18	[110]

## LIST OF ABBREVIATIONS

A2M: α2-Macroglobulin
ACAN: Aggrecan
ACI: Autologous Chondrocyte Implantation
ACs: Articular Chondrocytes
ADAMTS5: A Disintegrin and Metalloproteinase with Thrombospondin Motifs 5
AMPK: AMP-activated Protein Kinase
AP-1: Activator Protein 1
ATP: Adenosine Triphosphate
BMPs: Bone Morphogenetic Proteins
CDC42: Cell Division Cycle 42
COL10A1: Collagen Type X Alpha 1 Chain
COL11A1: Collagen Type XI Alpha 1 Chain
COL2A1: Collagen Type II Alpha 1 Chain
COL9A1: Collagen Type IX Alpha 1 Chain
DKK1: Dickkopf WNT Signaling Pathway Inhibitor 1
ECM: Extracellular Matrix
ENAH: ENAH Actin Regulator
FGF: Fibroblast Growth Factors
FGF-β: Fibroblast Growth Factor β
FRZB: Frizzled Related Protein
GTPase: Guanosine Triphosphate
HOX: Homeobox
IGF1: Insulin-like Growth Factor 1
IHH: Indian Hedgehog Signaling Molecule
IL-10: Interleukin 10
IL1β: Interleukin-1 Beta
IL-4: Interleukin 4
MACI: Matrix-assisted Chondrocyte Implantation
MDCs: Articular Chondrocytes Derived from Mesoderm
MMPs: Metalloproteinases
mTOR: Mechanistic Target of Rapamycin
N2K: ‘Nose-to-knee’
N-CAM: NC-cell Activated Matrix
NCDCs: Nasal Chondrocytes Derived from Neural Crest
NCs: Nasal Chondrocytes
NF-κB: Nuclear Factor Kappa-light-chain-enhancer of Activated B Cells
N-TEC: Nasal Chondrocyte Tissue-engineered Cartilage
OA: Osteoarthritis
PXN: Paxillin
Rac1: Ras-related C3 Botulinum Toxin Substrate 1
RhoA: Ras Homolog Family Member A
ROS: Reactive Oxygen Species
RUNX2: RUNX Family Transcription Factor 2
SOX9: SRY-box Transcription Factor 9
TGF-β: Transforming Growth Factor-β
TIMPs: Tissue Inhibitors of Metalloproteinases
TNF-α: Tumor Necrosis Factor α
VASP: Vasodilator-stimulated Phosphoprotein
